# AI-aided on-chip nucleic acid assay for smart diagnosis of infectious disease

**DOI:** 10.1016/j.fmre.2021.12.005

**Published:** 2021-12-28

**Authors:** Hao Sun, Linghu Xiong, Yi Huang, Xinkai Chen, Yongjian Yu, Shaozhen Ye, Hui Dong, Yuan Jia, Wenwei Zhang

**Affiliations:** a School of Mechanical Engineering and Automation, Fuzhou University, Fuzhou 350116, China; b Provincial Clinical College, Fujian Medical University, Fuzhou 350001, China; c Center for Experimental Research in Clinical Medicine, Fujian Provincial Hospital, Fuzhou 350001, China; d College of Mathematics and Computer Science, Fuzhou University, Fuzhou 350116, China; e Institute of Intelligent Manufacturing and Simulation, Fuzhou University, Fuzhou 350116, China; f Fujian Provincial Collaborative Innovation Center of High-End Equipment Manufacturing, Fuzhou 350001, China; g College of New Materials and New Energies, Shenzhen Technology University, Shenzhen 518118, China; h Sino-German College of Intelligent Manufacturing, Shenzhen Technology University, Shenzhen 518118, China

**Keywords:** Microfluidics, Deep learning, Polymerase chain reaction, Infectious disease diagnosis, Real-time predictive analytics

## Abstract

Global pandemics such as COVID-19 have resulted in significant global social and economic disruption. Although polymerase chain reaction (PCR) is recommended as the standard test for identifying the SARS-CoV-2, conventional assays are time-consuming. In parallel, although artificial intelligence (AI) has been employed to contain the disease, the implementation of AI in PCR analytics, which may enhance the cognition of diagnostics, is quite rare. The information that the amplification curve reveals can reflect the dynamics of reactions. Here, we present a novel AI-aided on-chip approach by integrating deep learning with microfluidic paper-based analytical devices (µPADs) to detect synthetic RNA templates of the SARS-CoV-2 ORF1ab gene. The µPADs feature a multilayer structure by which the devices are compatible with conventional PCR instruments. During analysis, real-time PCR data were synchronously fed to three unsupervised learning models with deep neural networks, including RNN, LSTM, and GRU. Of these, the GRU is found to be most effective and accurate. Based on the experimentally obtained datasets, qualitative forecasting can be made as early as 13 cycles, which significantly enhances the efficiency of the PCR tests by 67.5% (∼40 min). Also, an accurate prediction of the end-point value of PCR curves can be obtained by GRU around 20 cycles. To further improve PCR testing efficiency, we also propose AI-aided dynamic evaluation criteria for determining critical cycle numbers, which enables real-time quantitative analysis of PCR tests. The presented approach is the first to integrate AI for on-chip PCR data analysis. It is capable of forecasting the final output and the trend of qPCR in addition to the conventional end-point Cq calculation. It is also capable of fully exploring the dynamics and intrinsic features of each reaction. This work leverages methodologies from diverse disciplines to provide perspectives and insights beyond the scope of a single scientific field. It is universally applicable and can be extended to multiple areas of fundamental research.

## Graphical abstract

**Figure:**
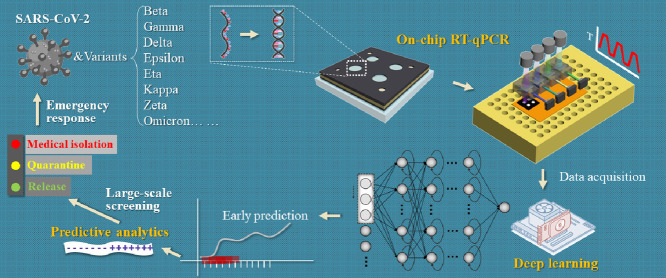

## 1. Introduction

**Fig. 1:**
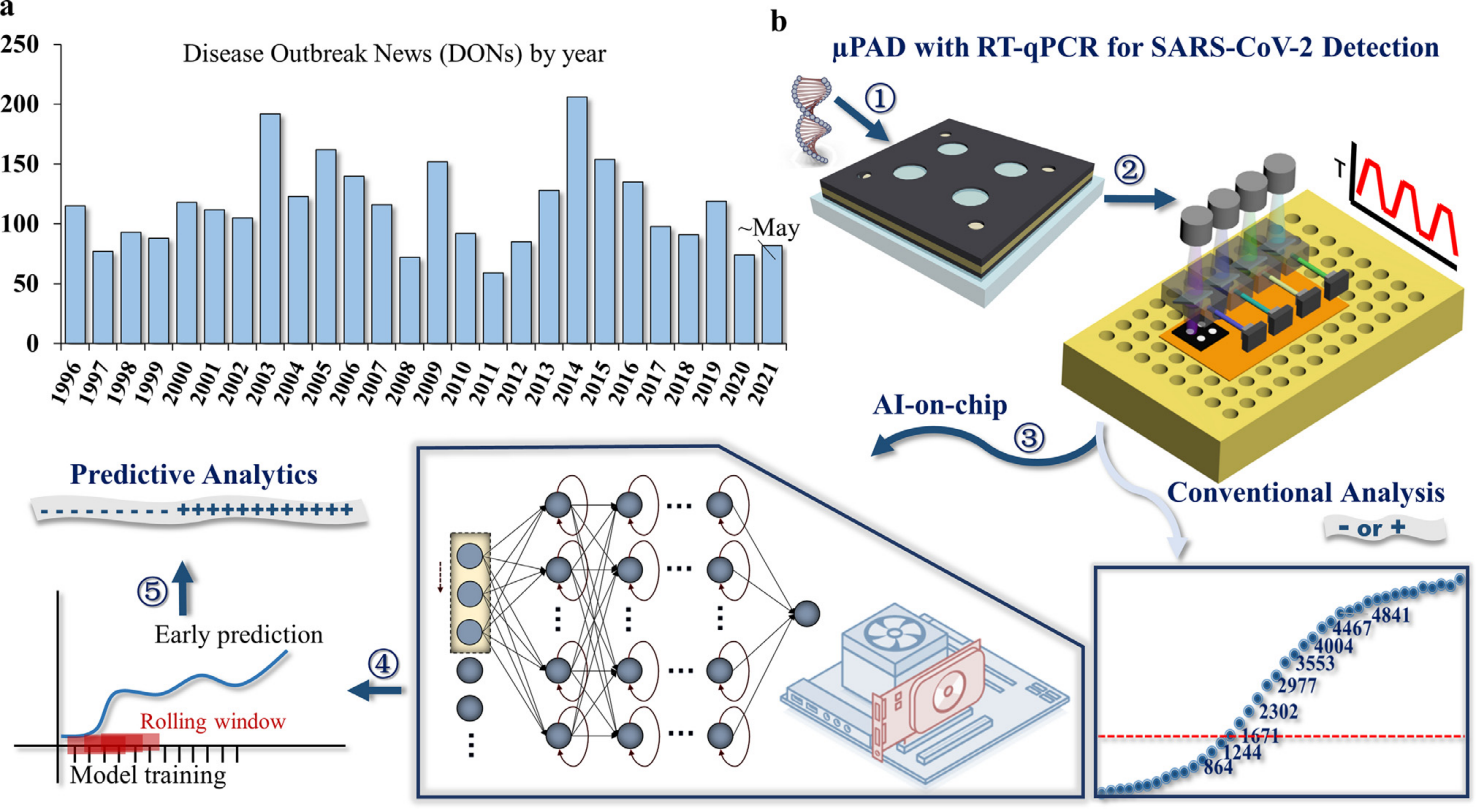
**Statistics of global infectious diseases and schematic of the proposed method.** (a) Disease outbreaks recorded by WHO. (b) Schematic of the proposed approach.

Throughout history, infectious disease outbreaks have ravaged humanity and destroyed civilizations. From the year 1996 to 2021, the world has witnessed about 2988 disease outbreaks (Fig. 1a) including SARS, Ebola, MERS, and COVID-19 [1]. Since 1970, more than 1,500 new pathogens have been discovered [2] and 51 to 67% of the world's population lacked essential health services according to the United Nations in 2019 [3]. Almost 100 million people are still in extreme poverty and surviving on just $1.90 or less per day [4]. On the other hand, even if medical countermeasures are available, infectious diseases will remain a great threat because of their rapid infectivity. Also, equitable access to effective public health measures is hard across the world [5]. The ongoing outbreak of COVID-19 has unmasked the underfunded nature and inequality of health care. COVID-19 has four interconnected traits: high reproduction number, a large number of asymptomatic or mild symptom cases, relatively long incubation period, and survival of the virus in some environments [6]. Reliable response to a new pandemic is mainly based on surveillance and detection, clinical treatment, prevention, and maintaining essential services [7]. Since developing new safe and effective medications often take a long time and viruses may mutate, containment and mitigation measures are the most key interventions to curb infections. Of these, containment in the early stages of the outbreak is critical for stopping transmission. Surveillance of at-risk people and identification of early case clusters based on polymerase chain reaction (PCR) have played critically important roles in sustaining the containment. PCR-based screening and mass (community or city-wide) testing have been routinely performed during the outbreak in mainland China [8]. These risk-based, large-scale screenings have successfully facilitated case finding and efficient restraining of epidemics and provided information for the government to safely reopen societies.

Although PCR is recommended by the WHO as the gold standard test for SARS-CoV-2, it is inherently laborious and time-consuming. Also, the turnaround time of a conventional PCR typically requires 4 to 6 h [9]. The commercially available plate-based PCR assays generally need to run 40 or more amplification cycles (∼1 h) to complete an analysis. While it should be noticed that the effectiveness of mass screening depends heavily on testing frequency and the speed of analysis. Strategies for more ‘smart surveillance’ of infectious diseases before their underlying large-scale emergence or re-emergence by the mutated virus are still needed [10]. In this regard, new technologies can be implemented to improve the mechanism and performance of PCR analytics, such as Artificial Intelligence (AI) and microfluidic paper-based analytical devices (µPADs).

AI, especially machine learning, has been developed with a broad range of applications for COVID-19 control and prevention [11,12]. For instance, enabled by large labeled datasets and GPUs, deep learning has shown excellent performance in machine vision tasks including image classification and object detection such as the analysis of chest radiographs (CXR) and chest computed tomography (CT) images [13], [14], [15]. Also, merely relying on initial clinical symptoms, AI helped predict COVID-19 test results [16]. Moreover, the growth and trend of the pandemic in countries worldwide have been forecasted [17]. Nevertheless, PCR analysis has been surprisingly neglected from machine intelligence. The dynamics of PCR are encoded in the time-oriented or chronological sequence of normalized reporter value (Rn) on a variable of fluorescent intensity [18]. However, the time series information is typically neglected by straightforward classifying the amplification curves into positive or negative readouts. In principle, machine intelligence can disregard the limitation of human cognition and is, therefore, a significant improvement in PCR data analytics. Recently, Moniri et. al. proposed a new amplification curve analysis method through a large volume of raw data by digital PCR and supervised machine learning [19]. To the best of our knowledge, AI-based dynamic analysis of PCR curves, which means making regression or prediction synchronously along with reaction, is barely studied. This capability will hold great potential to support current PCR-based studies in both clinical settings and fundamental research.

Microfluidics enables precise fluidic control and manipulation at a geometrically small scale (typically sub-millimeter) [20,21]. Compared with conventional microfluidics, microfluidic paper-based analytical devices (µPADs) have many promising merits: simple fabrication protocol and much less cost; relatively large surface-to-volume ratio due to porous nature of paper; fluid transport by capillary action without the need for external power sources [22,23]. Also, µPADs are portable and easy to use. The listed merits make µPADs particularly suitable to use in developing countries and areas short of medical resources [24,25]. In the past years, paper microfluidics have successfully performed sensitive assays that rival instrument-based nucleic acid amplification tests and provided precision diagnostics for pathogens with a fast turnaround time [26,27]. For instance, many µPADs have been developed and focused on LAMP tests of nucleic acids in infectious diseases [28,29]. Similarly, this technology could be leveraged for detecting SARS-CoV-2 nucleic acids [30,31].

In this work, we present a novel AI-aided on-chip approach by integrating deep learning algorithms with µPADs to detect RNA templates of the SARS-CoV-2 ORF1ab gene. The µPADs employ a multilayer structure and evaporation-preventive packaging technology, by which the device can be directly embedded into most qPCR instruments for data acquisition. Real-time PCR data are synchronously delivered to three unsupervised learning models with deep neural networks, including the stacked simple recurrent neural networks (RNN), the long short-term memory (LSTM) networks, and the gated recurrent units (GRU) layers. Of these, GRU is found to be the most effective and accurate for positive sample detection. Qualitative forecasting becomes available as early as 13 cycles or about 10 min. Accurate end-point value prediction of PCR curves can be obtained by GRU around 20 cycles with a mean absolute percentage error (MAPE) of 2.1%. Model parameter assessment study indicated that prediction accuracy improves along with the number of datasets. For negative samples, LSTM and GRU provide accurate qualitative predictions before 25 cycles. In addition, an empirical calculation method is proposed to determine the quantification cycle value (critical cycle) in real-time, which enables us to obtain the dynamics of PCR reaction much more rapidly without sacrificing testing accuracy. Various methodologies from precision manufacturing, instrument technology, molecular detection, and bioinformatics have been combined in this work to provide perspectives and insights beyond the scope of a single discipline. The presented approach is the first to integrated AI for on-chip real-time PCR data analysis. Furthermore, it demonstrates excellent compatibility between AI and the real-time characterization of biochemical reactions. Therefore, it is universally applicable and can be extended to various areas.

## 2. Principle, device, and experiments

### 2.1. Principle

The methodological framework of the approach is illustrated in Fig. 1b. Step 1 to 5 describes the workflow: sample collection, RT-qPCR in µPADs, model training and validation, early prediction on time series, and final output. Here, we selected synthetic RdRp gene (RNA-dependent RNA polymerase gene) in the open reading frame ORF1ab region of SARS-CoV-2 as a target (Fig. S1) and used a set of primer and probe to detect its gene sequence. This operation was compatible with the recently developed extraction-free SARS-CoV-2 RT-PCR. Synthetic nucleic acid, negative control (NC), substrate mix, and enzyme mix were introduced sequentially onto µPADs for on-chip tests. The µPADs were installed in a commercial qPCR instrument, which was then used for reliable data acquisition. The excellent compatibility between our device and the commercial instrument also indicated the wide applicability of the device and method. Unlike conventional RT-qPCR which provides one-off results including positive/negative readout, Cq (quantification cycle) at the end of a whole test, the AI-on-chip approach allowed real-time analysis during the amplification cycles. Values of fluorescent intensity during qPCR were recorded and real-time fed into the networks for model train and test followed by prediction.

Classic machine learning employs algorithms such as the k-nearest neighbor, support vector machine, and decision tree for feature learning, model construction, and model training. Although these classical models have been widely used in performing multiple tasks including classification and pattern recognition, they often require structured data sets and are dependent on human intervention to learn. For instance, the information presented in PCR curves, which includes slope, mean, variance, standard error, minimum and maximum values, as well as other known features, can be intuitively gathered and processed by a human. Despite being theoretically possible, the preprogrammed feature extraction and filtering process are time-consuming and will be inconsistent by subjective experience. Additionally, the effect of these correlation mechanisms on the final results may not be readily coded in advance.

Deep learning allows autonomous data processing towards sophisticated and nonlinear feature abstraction through a cascade of layers of neural networks, instead of inputting the optimum feature representation by expert knowledge [32]. Here, we utilized RNN, the algorithm employed by Google's voice search and Apple's Siri, for qPCR sequential data analysis. In parallel, the most well-known subsets of RNN, LSTM, and GRU have been used for improving model performance. Using these deep neural networks, features of PCR curves can be automatically extracted followed by real-time model training without being explicitly programmed. Essential fundamentals of RNN, LSTM, and GRU can be found in literature [33,34]. In brief, all three networks take the present and the past as input sources for determining the output or response to new data. The decision made by these models at time step t-1 influences the decision at time step t. Different from classic (or "vanilla") RNN, LSTM is composed of a cell containing an input gate, an output gate and a forget gate. By adding the gating mechanism, information can be stored in, written to, or read from a cell which is helpful to partially avoid the vanishing gradient problem. Similarly, GRU keeps the mechanism by deploying reset gate and update gate but excluding output gate. Performances of the three models on predictive analysis of qPCR have been studied in later sections.

### 2.2. Device design and fabrication

**Fig. 2:**
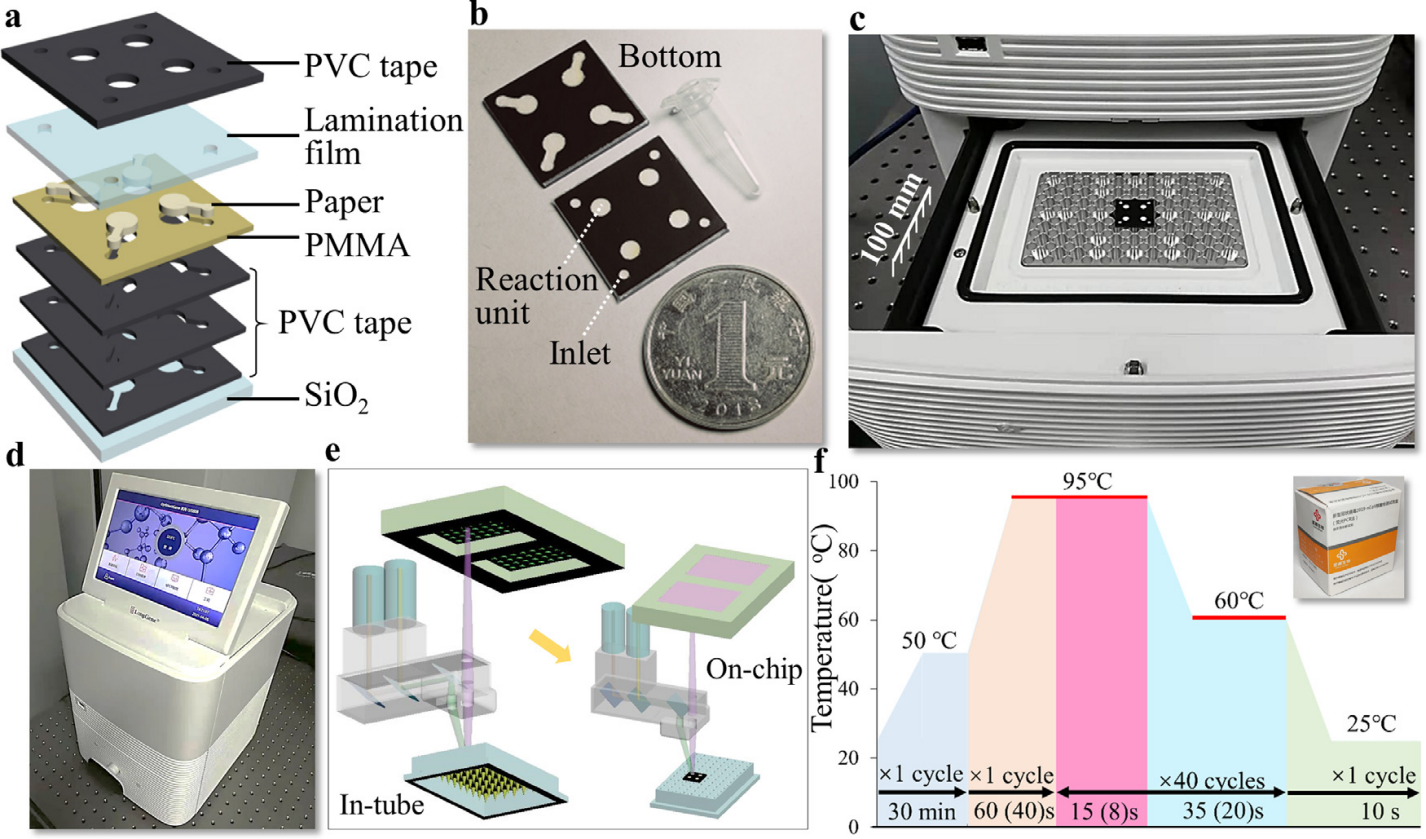
**Chip design, fabrication, and experimental set-up.** (a) Design of the paper chip. (b) Packaged chip prototypes. (c) Chip in a 4-channel multiplex qPCR (quantitative polymerase chain reaction) machine. (d) Image of the machine. (e) The schematic of fluorescence detection. (f) Program operation panel (time duration in brackets is set for on-chip tests).

The architecture of a µPAD contains seven layers (Fig. 2a). Glass slide containing 97% silica was attached by graphite thermal conductive adhesive at the bottom and used as a solid substrate (not shown in the schematic and image). Since the thickness of a paper cannot be neglected, three non-transparent layers of polyvinyl chloride (PVC) were coated together on the substrate to create dumbbell-shaped hollow wells, which were used for fixing paper fluidic layers. Then, the paper layers were inserted into the wells. Double-sided adhesive polymethyl methacrylate (PMMA) was used as a connecting layer. A thin film of PVC with thermosensitive gel (ethylene-vinyl acetate copolymer, EVA) was laminated on top of the paper. The PMMA layer strengthened the binding of upper with lower PVC films. Finally, another black-colored PVC layer was placed on top of the assembled device for reducing background noise from ambient lighting. For proof-of-concept study, the current chip allows for parallelized testing of up to 4 samples and can be further increased as needed. Circle-shaped paper layers in the peripheral region were designed as reagent inlets. Circles distributed in central (fully covered by lamination film) were designated as reaction units. The position and size (3.5 mm in diameter) of the reaction units were rigidly designed so that the center of the units aligns with the light focus and the heat sink of the qPCR instrument. The overall dimension of the portable device is 20 mm × 20 mm × 1.6 mm in length, width, and height, respectively. Additionally, the total cost of an assembled device is limited to below 1.6 RMB. The layout design of each layer was completed in the vector graphics software Adobe Illustrator. More details can be found in Fig. S2.

Fabrication of μPADs employed laser cutting technology. The distance between lens and workpiece of the machine (JK-4060, Jingke Company) was 60.0 mm. The power used for cutting paper, PMMA, and PVC were 14.5, 15.0, and 15.0 W, respectively. The line speed of the cutter was 12.0 mm/s. Packaging of the chip was completed by combining both mechanical force and heating lamination. The pressure-sensitive adhesive film was placed in between the device layers except for both PVC/EVA-paper layers, and mechanical forces were applied to strengthen the bonding. Through holes were drilled on the PVC/EVA film in advance. Then, the patterned film was used for single-side paper lamination (YE381, Soonye Tech. Co Ltd) at a temperature of 130 °C. The lamination effectively eliminated chip reagents' evaporation during the thermal cycling of PCR. A scanning electron microscope (by FEI Nova NanoSEM 230, Thermo Fisher) was used for morphology analysis of fabricated devices. The total expense of a μPAD is around 0.24 USD (Table S1), thus making the chip economically applicable in underdeveloped areas.

### 2.3. Materials and procedure

Surface RNase Erasol was purchased from Phygene® Biotechnology Co, Ltd (Fuzhou, Fujian, China). Whatman® Grade 5 filter papers were purchased from GE Healthcare Life Sciences (Pittsburgh, PA., USA). PVC pressure adhesive films were obtained from HUACHEN Paper (Jinhua, Zhejiang, China). Laminating films (PET with EVA) with a thickness of 0.03 mm were obtained from ZhongWei Technologies (Fuzhou, Fujian, China). Double adhesive PET films were purchased from BOSSRON (Guangzhou, Guangdong, China). EVA hot melt glue stick was obtained from ZHONGHA (Jinhua, Zhejiang, China). COVID-19 Nucleic Acid Diagnostic Kit (PCR-Fluorescence Probing, NIRC20203400064) was obtained from Sansure Biotech Inc. (Changsha, Hunan, China).

The COVID-19 testing kit consists of four reagents: Substrate Mix, Enzyme Mix, synthetic RNA templates, and NC. Main ingredients of Substrate Mix contain premiers (4.62%), probes (1.15%), dNTPs (3.85%), MgCl_2_ (0.77%), RNasin (0.48%) and PCR buffer (89.13%). Enzyme Mix contains both RT enzyme (62.5%) and Taq enzyme (37.5%). The Positive Control is provided within vitro transcriptional RNA which contains target genes (ORF1ab, N gene) and internal standard gene fragments (RNase P), whose fluorescein of hydrolysis probes is FAM, ROX, and HEX, respectively. Negative Control contains saline only. 83 samples of synthetic gene templates of SARS-CoV-2 were used following the protocol recommended by the manufacturer.

The experimental procedure started with placing the diagnostic kit reagents at room temperature to allow them to equilibrate, followed by a vortex step at a speed of 3000 rpm for each reagent. Then, substrate mix (26 μL) and enzyme mix (4 μL) were pipetted into tubes for premixing by centrifuging at a speed of 2000 rpm for 15 s (MC-12plus, JOAN LAB Equipment Co., Ltd). Next, the sample containing synthetic RNA templates (10 μL) and NC were separately introduced to the tubes containing 30 μL of PCR master mix. 1.5 μL of each mixed reagent was introduced to the inlet of the μPAD. To eliminate evaporation of on-chip reagents, we sealed the inlets using hot melt glue. Then, the chip was transferred to a qPCR instrument (Q2000B, LongGene Scientific Instruments Co., Ltd.). Different from the in-tube tests, heating time for denaturation (at 95 °C), annealing, and elongation (at 60 °C) were 8 and 20 s for the on-chip test, comparing with 15 and 35 s for the in-tube test. This meant the total run time for a conventional 40-cycle conventional qPCR test was reduced by more than 880 s using the on-chip method.

## 3. Results and discussion

### 3.1. Data acquisition and evaluation

**Fig. 3:**
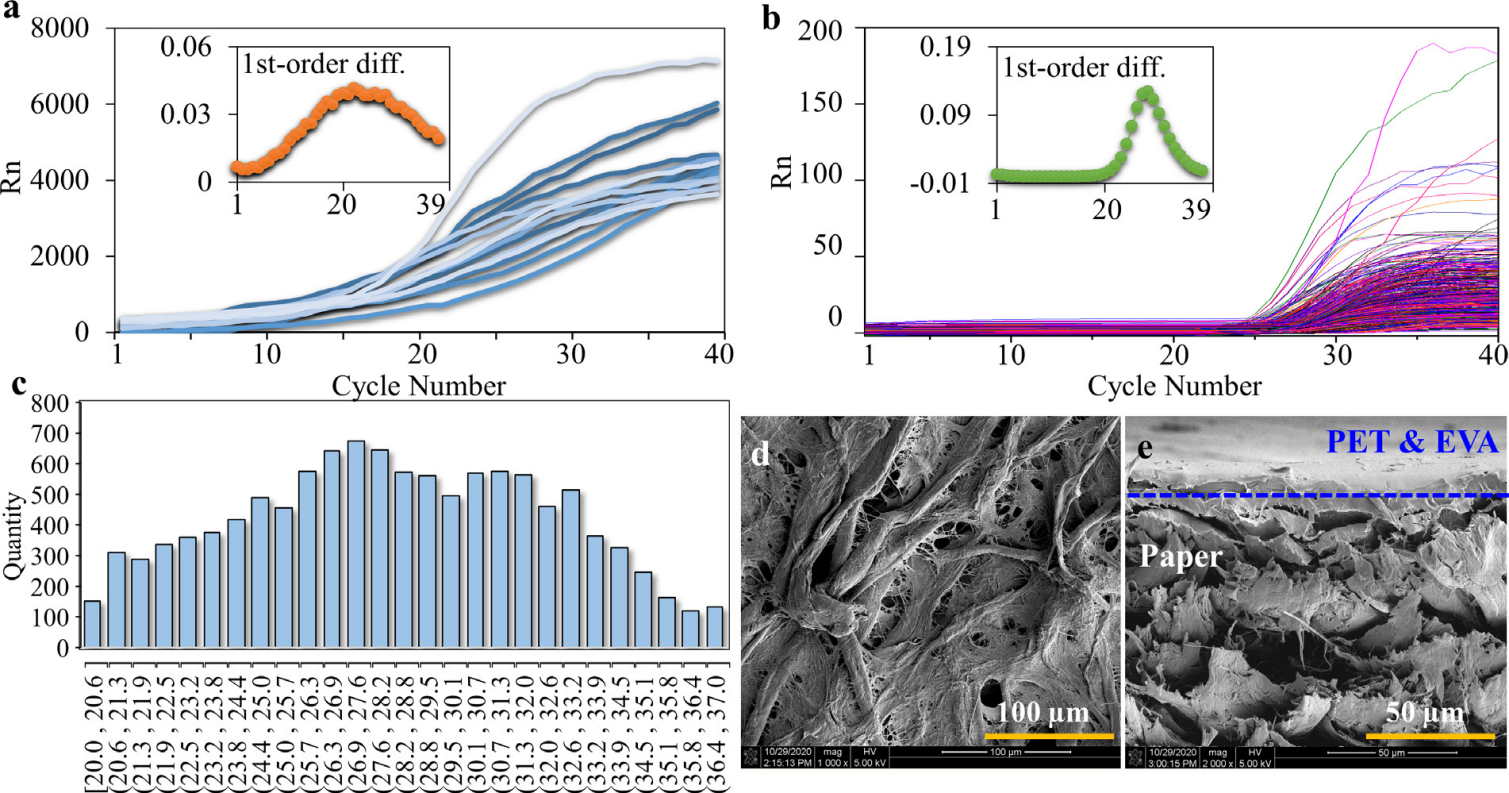
**Data evaluation and micromorphology analysis of cellulose paper.** (a) A group of qPCR curves obtained by on-chip tests. (b) 11388 amplification curves obtained from the clinical lab of Fujian Provincial Hospital. Insets of (a) and (b) describe the first-order difference value of the curves. (c) Cq distribution of the curves. (d) Scanning electron microscope (SEM) image of paper material. (e) SEM image of the cross-section of a laminated paper chip.

The on-chip dataset contains 83 data plots (during a period of 16^th^ April 2021 to 5^th^ June 2021) of synthetic gene templates of SARS-CoV-2 using the protocol recommended by the manufacturer (Fig. 3a). For real-time data acquisition, the on-chip amplification data were automatically written into a .txt file which was then read and processed by the AI program using the same computer. LongGene Scientific Instruments, the manufacturer of the PCR instrument provided the technical support for the real-time data transmission. Datasets of qPCR in a time-series format from the Center for Experimental Research in Clinical Medicine (CERCM) of Fujian Provincial Hospital (during a period of 4^th^ August 2020 to 3rd November 2020) and on-chip tests were also assessed. The in-tube dataset (Fig. 3b) contains 11388 nucleic acid amplification curves. Cq values of these plots were mainly distributed within a range of 20.0 to 37.0 (Fig. 3c).

On the basis that a value from data X at a given time is related to the previous values, the series of values can be described as $X=\{x_{1},\;x_{2},..x_{t}\}$. Herein, (t) is the most recent value. This deep learning model aims to predict (t+N) from historical values containing sequence data features, where N is named as prediction interval (PI). PI is a range of values for future prediction, and it is likely to be far more useful in decision-making than an individual number. Using the two datasets, for preliminary analysis, we calculated either the dynamic slope or the first-order difference (FOD) of the Rn value at each cycle point: *Δx_t_ = x_(t+1)_-x_(t)_*. Herein, t and x are cycle numbers and Rn values. Mean values of FOD at each cycle of 83 (or 11388) curves were plotted as shown in the inset of Fig. 3a (or Fig. 3b). Both of the FOD curves are overall bell-shaped, which coincides with the sigmoid curves of the original amplification data. For in-tube tests, the FOD curve is sharper and the values increase rapidly after 22 cycles and reach the peak value of 0.125 at the 29^th^ cycle. While, for on-chip tests, the FOD curve starts to climb as early as the 3rd cycle and maximizes with a value of 0.042 at the 23^rd^ cycle. We attribute the early rise of FOD values to the paper material. The fibrous nature of the paper material provides a high surface-to-volume ratio (S/V), which in turn enhances the detecting performance. Specifically, paper porous microstructures create abundant reaction sites and opportunities (Fig. 3d, e), and therefore significantly improves reaction speed. Also, compared with the stereo in-tube reaction, fluid transport in the in-plane dimension of paper chips dominates so that more fluorescent reporters can be delivered onto the top surface, directly below the light source, which improves detecting sensitivity (smaller limit of detection). Finally, it has been proven that a higher S/V may induce a wider linear range for fluorescein on microscale. The fluorescence technique can further improve detection performance over traditional colorimetric detection. Notably, the background fluorescence of the paper material may cause complications during PCR tests. However, for this study, the merits of the paper material outweigh the background issue.

The symmetrical range of the FOD curve for the in-tube tests is determined to be 18 cycles with a respective value increased from 0.008 at the 23^rd^ cycle to the maximum and then returned to 0.008 at the 40^th^ cycle. The corresponding range for on-chip tests is found to be 29 cycles, starting with a value of 0.018 at the 12^th^ cycle and returning to a value of 0.019 at the 40^th^ cycle. The rise of the FOD curve for the in-tube test happened in a much later time than the on-chip test. Therefore, effective forecasting of on-chip tests has more practical merits in shortening the turnaround time of PCR assay. Based on the premises, the on-chip tests were confirmed to be adopted for predictive analytics.

### 3.2. Deep learning pipeline

Python environmental (version: 3.8.5) and TensorFlow (version: 2.3.0) were employed to create deep learning models. Deep neural networks are usually hindered from time series forecasting since the data are typically nonlinear and highly dynamic [35]. Here, we constructed a deep learning pipeline to automate the workflow. The procedure of the pipeline includes the processing of data augmentation and normalization, dataset splitting followed by model training, testing, and time series prediction.

**Fig. 4:**
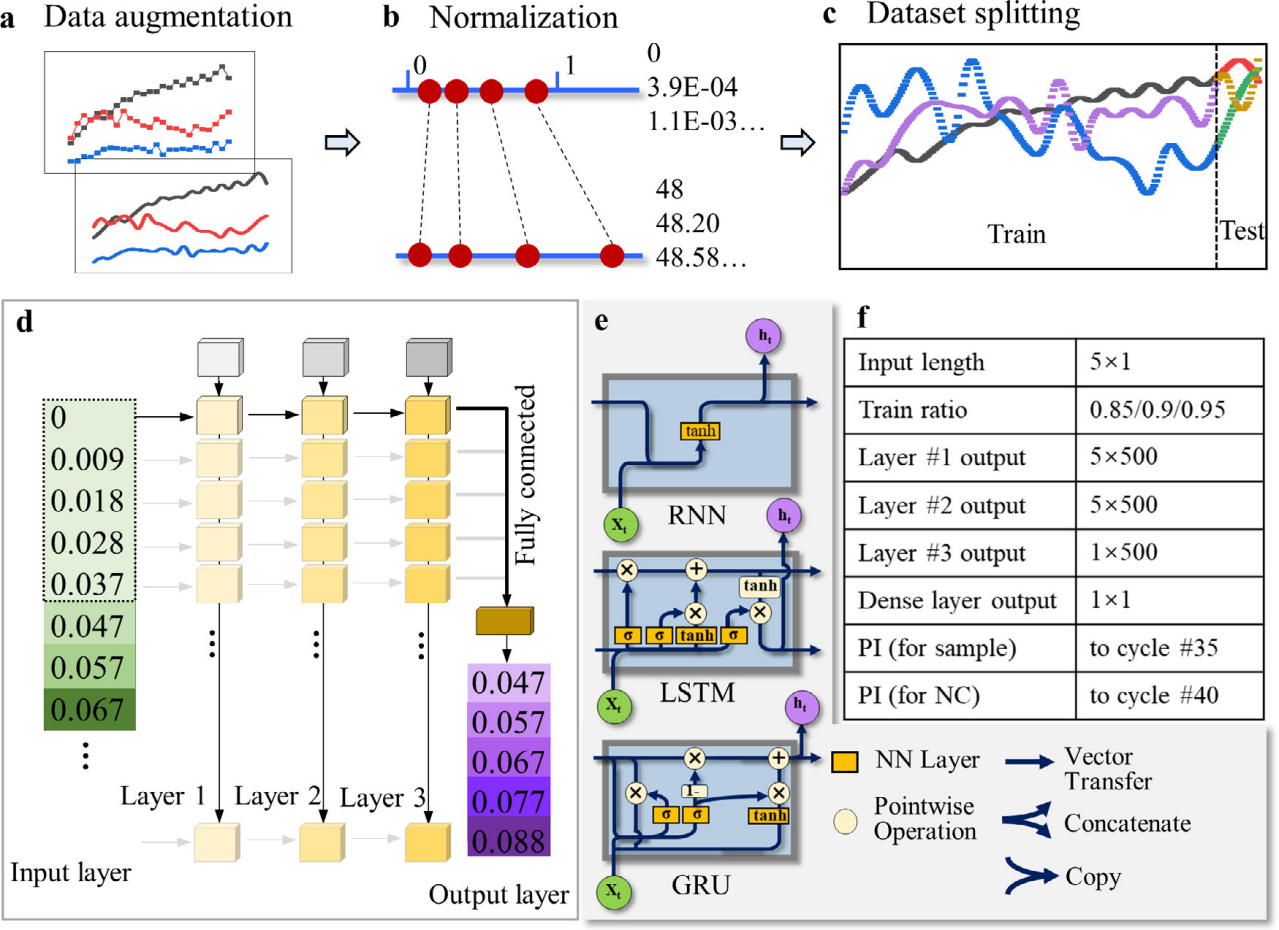
**Deep learning pipeline.** (a) Data acquisition and augmentation; (b) Normalization; (c) Splitting; (d) Model training, test, and prediction. (e) Internal structure of the three networks. (f) Parameters of the network.

Before feeding the data into the deep learning models, data augmentation was firstly performed (Fig. 4a). This pre-processing step has shown efficiency in improving model performances in general and is popular in computer vision study, but not for time-series data processing. In this work, we employed interpolation, which had been proven to be effective for improving the performance of deep learning models [36] to perform time-series data augmentation. Quadratic Bézier curve fitting was selected as the interpolation method after comparing with linear and cubic interpolation methods. Using the identical dataset, the quadratic interpolation was the most robust, efficient, and simple, and thus was adopted in this work. A set of data points was interpolated between adjacent cycle numbers following the equation below:

$$P_{i}\left(t\right)=\frac{1}{2}{\left(t-1\right)}^{2}P_{i}+\frac{1}{2}\left(-2t^{2}+2t+1\right)P_{i+1}+\frac{1}{2}t^{2}P_{i+2},0\leq t\leq 1$$

In addition, data normalization, which affects the accuracy and generalization of time series forecasting, is a necessary and important pre-processing technique for deep learning [37]. We scaled the data to a range of [0, 1] using the Min-Max normalization method (Fig. 4b) expressed as: *x*=(*x*-*x*_min_) / (*x*_max_-*x*_min_). Subsequently, the time series dataset was divided into train set and test set to fit the machine learning model and evaluate the trained machine learning model, respectively (Fig. 4c). The dataset splitting ratio was modulated from 0.85 to 0.95, which was adjusted according to different stages of PCR tests. Specifically, for below 10 cycles, the ratio was set to be 0.85; between 11 and 15 cycles, the ratio was 0.9; beyond 15 cycles, the ratio was 0.95.

The selected data augmentation and normalization methods are typical and can be readily used for rapid data processing. The pre-processed data was then applied to neural networks for model training. RNN is well-suited for solving time series prediction issues [38]. Compared with other commonly used neural networks that are formed by multilayer perceptron and can only map input data to target vectors, RNN can trace back to historical inputs. A back propagation algorithm was adopted for training RNN. A typical RNN is based on a theory that *h_t_ = f(x_t_, h_t−1_)*, which introduces a recurrent structure. By stacking multiple RNNs on top of each other, the performance can be further boosted. Therefore, three hidden layers of vanilla RNN, LSTM, and GRU where each layer contains multiple cells were employed (Fig. 4d, e). The applicability of RNN has been mostly limited by gradients vanishing or exploding issues. LSTM networks are a subset of RNN with an additional input gate, an output gate and a forget gate added to each standard cell. The three gates regulate the flow of information into and out of the cell. By this regulating mechanism, LSTM can partially solve the vanishing gradient problem. Similarly, GRU follows the mechanism by deploying a reset gate and an update gate but excluding the output gate. GRU has shown better performance on smaller and less frequent datasets. Up to date, there have been limited reports on the interdisciplinary study of PCR and RNN. Most recently, it has been revealed that a combination of RNN with biological features outperforms other methods for activity prediction of RNA design [39]. Also, prediction of PCR amplification based on primer and template sequences was achieved using RNN [40]. As laboratory studies, both of the reports were not focused on dynamically predicting the end-point output of PCR by previous data along the amplification curve. This ability, however, will be much attractive and practical for clinical settings. In this work, for the first time, RNN, LSTM, and GRU acquire knowledge straightforwardly through the training process and are applied to predict the Rn values of PCR tests. Parameters of the neural networks are illustrated in Fig. 4f. Input length indicates the number of data points in sequence fed into the deep learning model. Using the open-source software library Keras, stacked RNN, LSTM and GRU were constructed. Within the network, each of the three hidden layers contained 500 neurons. Linear activation function was adopted by vanilla RNN layers. Hyperbolic tangent activation function or Tanh was used for LSTM and GRU layers. Mean squared error (MSE) was used as a loss function and adaptive moment estimation was selected as the optimizer. A dense layer connected all the neurons in the third RNN/LSTM/GRU layer. PI for unknown sample tests was set to be 35 cycles following a common observation in qPCR tests. For negative control tests, the PI was 40 cycles for adequately detecting the background signal which may affect final interpretation.

### 3.3. Accuracy

qPCR curve in a sigmoidal shape is the fluorescence response to the growth of amplified product during the reaction process. Conventional PCR analytics primarily focuses on quantitative responses involving cycle number determination. Analysis based on Cq (or Ct used by machine manufacturers and clinicians) provides a quantitative assessment by focusing on the exponential growth region of the amplification curve. However, Ct refers to a real-time predictive value whose scientific accuracy or clarity is heavily dependent on PCR instruments. Generally, the threshold for obtaining Ct values is set either based on an internal quantitation standard (by instrument manufacturer) or empirical evaluation. Typically, a qPCR instrument software sets the threshold at 10 times the standard deviation (SD) of the fluorescence value of the baseline. However, the manufacturer also emphasized that the threshold can be set at any point in the exponential phase of PCR. Furthermore, a baseline is defined as the initial cycles of PCR during which the variation in fluorescence signal (usually from the 3^rd^ to the 15^th^ cycle) is insignificant. Limitations of the traditional method lie in (1) the cycle range of baseline. Specifically, the baseline can only be assessed after 15 cycles, and thus an earlier Cq value cannot be obtained in real-time until the 15^th^ cycle (even though the end cycle value can be smaller than that of the 15^th^). (2) Processing of anomalous signals. Provided that the threshold is low, the presence of signal anomalies (may be due to bubbles or evaporation) makes the distinction of Cq values between a false threshold crossing and signal response difficult. In some cases, even minute errors in the baselining process can cause false signals to cross the threshold. (3) Variation of Cq values. Based on recent literature, Cq values of SARS-CoV-2 testing varied greatly between and within methods, sometimes even within a single test using the identical instrument [41]. Therefore, the difference in Cq values for the same target cannot be simply neglected. By employing the deep learning models, the dynamics of the amplification reaction process can be directly measured. Features hidden in time-series amplification data were automatically extracted and studied without requiring user intervention. Therefore, dynamic mechanisms of the PCR reaction can be explored in much more detail compared with human cognition.

For a proof-of-concept study, a group of PCR curves consisting of three positive samples and three NC tests was selected. Firstly, time-series datasets were kept as a reference for algorithm comparison. Early predictions of positive time-series data were made starting from the 21^st^ cycle for samples #1 and #2, and from the 22^nd^ cycle for sample #3. Predictions on all the negative data were started from the 25^th^ cycle. For the positive samples, true values of Rn at the end-point cycle using µPADs were obtained to be 3293.87, 4074.67, and 6946. For the three NCs, true values of Rn at the end-point cycle were 801, 2682.93, and 531. Using vanilla RNN, LSTM, and GRU algorithms, 35-cycle amplification for a positive sample and 40-cycle amplification for NC tests were predicted. Train/test loss plots of the three models using the mean squared error (MSE) function are shown in Fig. S3. All MSE values decreased with model iterations until reaching a saturation value.

**Fig. 5:**
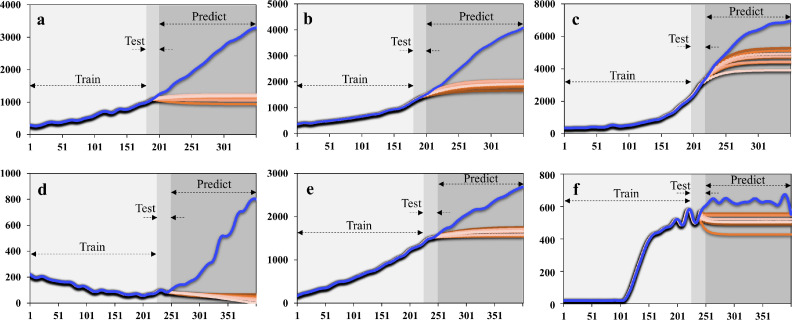
**The real (in light blue) and predicted (in gradient orange) curves consisted of trained, tested, and predicted values by the recurrent neural networks.** (a) to (c) from positive samples. (d) to (f) from negative samples.

Specifically, mean Rn values at the point (the 35^th^ cycle for positive sample) predicted by vanilla RNN (Fig. 5) were 1142.27, 1911.48, and 4583.40. Mean Rn values at the same point (the 40^th^ cycle for the negative control) predicted by RNN were 35.76, 1661.07, and 538.97. We used the MAPE to evaluate accuracy:

$$MAPE=\sum_{t=1}^{n}\left|\frac{observed_{t}-predicted_{t}}{observed_{t}}\right|\times \frac{100}{n}$$

The values were 20.47%, 14.76%, 9.94% for the three positive samples, and 29.53%, 9.47%, 14.79% for NCs by vanilla RNN-based prediction. The forecasted trends by RNN were inconsistent with the true values. Therefore, it can be concluded that the accuracy of the vanilla RNN method was unacceptable.

**Fig. 6:**
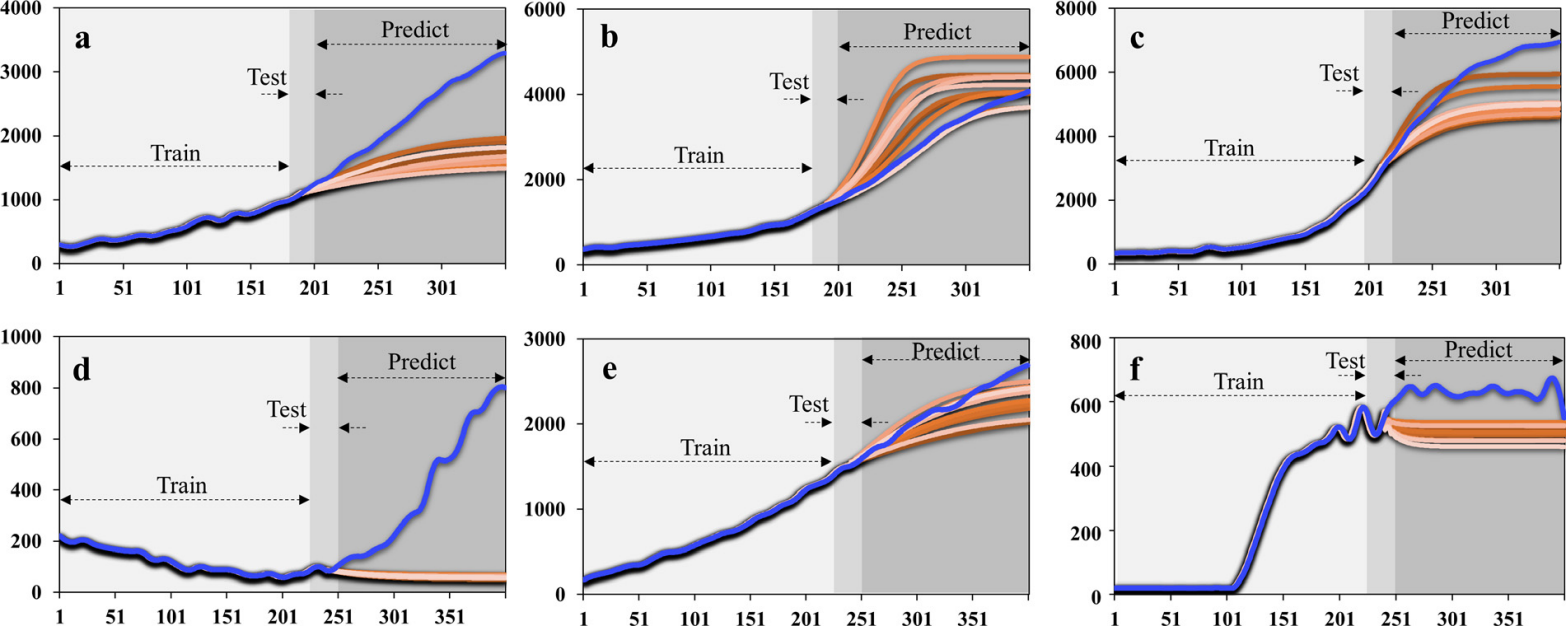
**The real (in light blue) and predicted (in gradient orange) curves consisted of trained, tested, and predicted values by the long short-term memory method.** (a) to (c) from positive samples. (d) to (f) from negative samples.

By contrast, using the identical datasets, mean Rn values at the end-point predicted by stacked LSTM (Fig. 6) for the positive samples were 1691.07, 4194.63, and 5029.1, and 60.45, 2287.7 and 482.57 for the NCs, respectively. The corresponding MAPE values were 13.77%, 8.58%, 7.91% for the three positive samples, and 29.18%, 2.25%, 14.98% for NCs. The dynamic trends of the forecasting curves were analogous to the true plots of positive samples #2, #3 (Fig. 6b, c) and negative control #2, #3 (Fig. 6e, f). However, the prediction made based on the LSTM methods showed rather a large discrepancy from the true curves for both positive sample #1 and NC #1. Notably, for this case of NC#1, the deep learning model predicted the output to be negative which coincided with the real results. A potential explanation is that some of the reagents may have evaporated in the first 20 cycles, as indicated by the true data curve (Fig. 6d). After reagents were introduced to the paper chips, the background fluorescence of the paper was known to be suppressed. Nevertheless, if the device was improperly packaged, the thermal cycling process could cause the paper to dry due to evaporation, thus inducing background fluorescence. After the paper completely dried out, the fluorescent signal increase rapidly due to the paper background intensity. Furthermore, the magnitude of this false signal was not on the same scale as obtained from actual PCR tests. Consequently, we can conclude that the predictive performance by the LSTM algorithm was better than that of vanilla RNN but still has room for improvement.

**Fig. 7:**
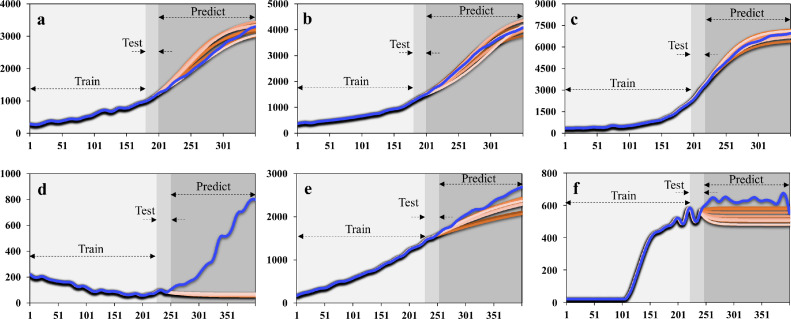
**The real (in light blue) and predicted (in gradient orange) curves consisted of trained, tested, and predicted values by the gate recurrent unit method.** (a) to (c) from positive samples. (d) to (f) from negative samples.

Finally, Mean Rn values at the end-point predicted by stacked GRU (Fig. 7) for sample and NC tests were 3239.87, 4110.54, 6821.57, and 63.92, 2302.53 504.56, respectively. Correspondingly, MAPE values were 3.57%, 1.18%, 1.65% for the three positive samples, and 29.1%, 3.6%, 13.25% for NCs by GRU-based prediction. The dynamic trends of predicted curves were in good agreement with the true plot for all positive and negative samples (Fig. 7) except the NC #1 (Fig. 7d). The offset phenomenon in Fig. 7d has been discussed above. Here, we noticed a linearly increasing trend in the true plot as shown in NC #2 test. Although the end-point values were relatively high compared with other NC tests, they were still well below the end-point values of the sample tests. Therefore, the monotonically increasing of signals was not caused by nucleic acid amplification. This downward trend was also found in the other two NC tests using the GRU model, which could be easily construed as a negative control. Thus, based on the deep learning prediction method, anomalous data from pseudo reactions were more likely to be recognized as a negative output. To sum up, the GRU model was highly accurate for quantitative analysis and was well-suited for interpreting information from PCR tests. For qualitative analysis, the deep learning model can also make an accurate prediction in a binary format. Measurements of prediction accuracy using MAPE, MAE, and SMAPE are shown in Table S2.

As seen in Fig. 7, the standard deviations (SD) of predicted data were 146.89, 192.17, and 271.97 by positive testing results, and 5.97, 119.66, 35.47 by NC tests. After 10 repeated tests, all SD values were lower by more than an order of magnitude of the output, indicating excellent reproducibility. The robustness of measurements was also evaluated by interpreting results regardless of the experiences of the manipulator performing or reviewing the test. As a result, the machine intelligence aided on-chip qPCR has the potential to achieve highly automatic and robust diagnostics.

### 3.4. Early prediction

In general, accuracy is the most significant evaluation criterion. At the same time, rapid screening and detection of a pathogen at the beginning of an unknown infectious disease is critical. The most attractive merit of AI-aided on-chip qPCR is the rapidness or less turnaround time for each assay. For instance, for SARS-CoV-2 detection, no quantitative assays have yet received Emergency Use Authorization (EUA) by the Food and Drug Administration (FDA). There is also no international standardization available, which is necessary for quantitative assays. In this scenario, predictive analytics should target an optimum balance between earliness, which is an ability to provide a decision early, and accuracy.

**Fig. 8:**
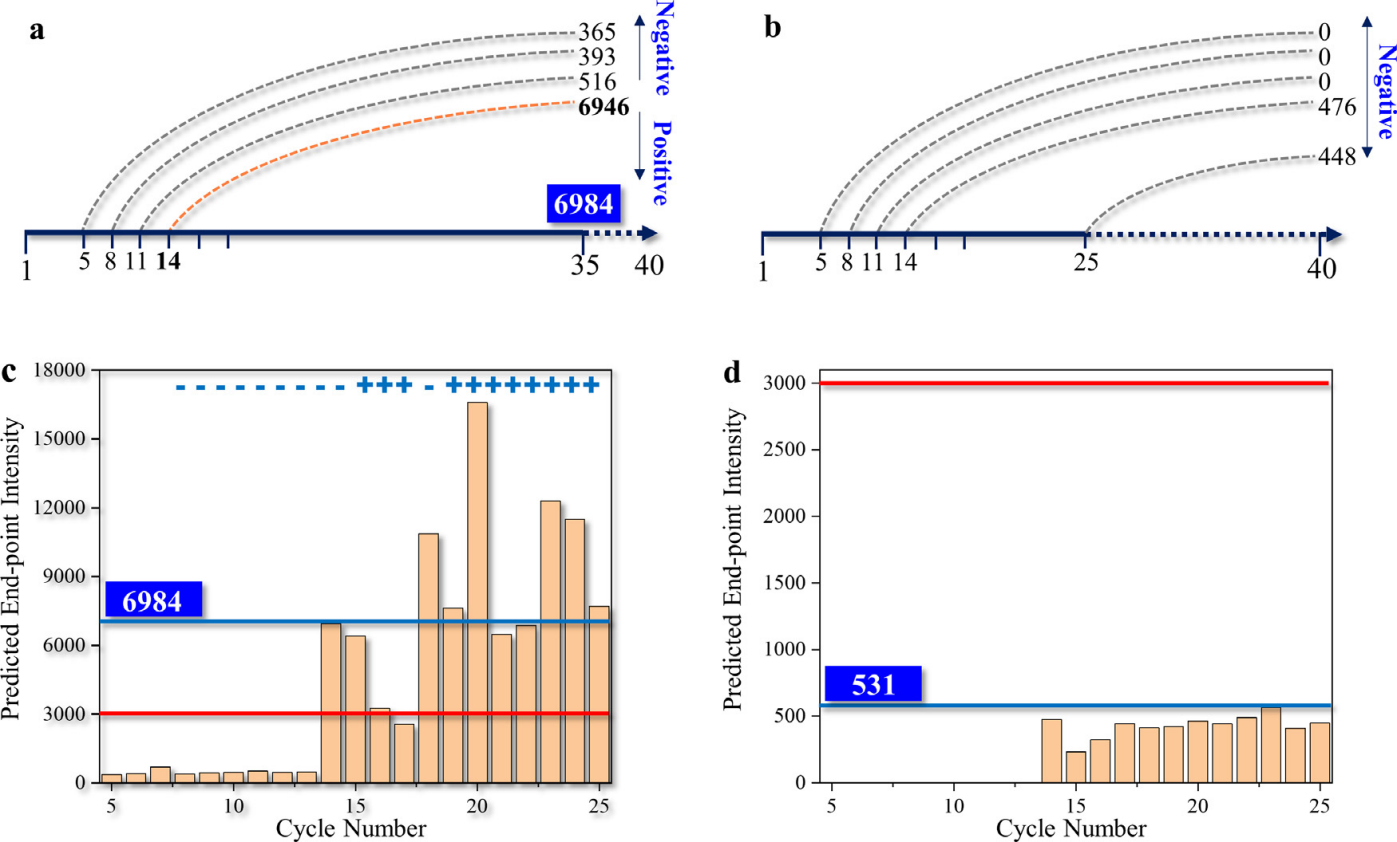
**Early prediction tests.** (a) Correlation of early cycle number with predicted intensity at the end of the 35^th^ cycle (positive sample). (b) Correlation of early cycle number with predicted intensity at the end of the 40^th^ cycle (negative sample). (c) Dynamic prediction based on medium cycle number and quantification cycle-based criterion. (d) Dynamic prediction based on medium cycle number (#25) and negative determination.

In the above accuracy study, we employed deep neural networks for predicting data in the latter sequence from a given cycle number (21, 22, or 25). Here, for revealing the earliness of prediction by this approach, we took positive sample #3 which had a nearly standard sigmoid shape and NC #3 for demonstrative study. Since the end-point value reflecting final reaction yields is a critical indicator for the determination of positive or negative results, the correlation of early cycle numbers with final fluorescent intensities was obtained as shown in Fig. 8. For the positive sample (Fig. 8a, c), predicted intensity at the endpoint (the 35^th^ cycle) was consistently lower (∼1000) compared with the intensity value before the 13 cycles. Then, the predicted value raised rapidly when the 13^th^ cycle data was fed to the train/test dataset of the deep learning model. The average output value was 6406.02 which was comparable to the true value of 6984. After this, the predicted value gently decreased for a short period ranging from the 15^th^ to the 17^th^ cycle followed by rising again. When data from the 20^th^ and 21^st^ cycles were entered, predicted values were found to be in good agreement with the true data.

Based on the empirical data, a criterion is defined for qualitative prediction: a sample can be determined as positive when the intensities at a cycle and the subsequent two cycles exceed a threshold. Here, we use Rn = 3000 as a threshold which is reasonable considering the data shown in Fig. 3a. Following the criterion, the sample can be determined as positive at the end of the 13^th^ cycle, thus effectively shortening the qPCR time duration by 67.5%. Considering the time cost of an on-chip PCR was limited to below 40 min, the turnaround time of AI-aided microfluidic assay was merely about 12 min. The applicability can be extended further because the dynamic or real-time nature of AI-based prediction will certainly offer knowledge before the end of reactions. Moreover, for a quantitative study, the criterion can be explained as: a cycle number can be determined as the critical number when the intensity at the cycle and the subsequent two cycles are all above the threshold. Output values predicted at the critical cycle were comparable to the true data. The deep learning model can be further improved by training more datasets. Keeping experimental settings and operation procedures consistent, target loads in different reaction units can be compared using the discussed critical cycle values. Therefore, the quantification of intragroup assays can be performed. Similarly, the cycle-dependent output of NC predicted by GRU-based networks is presented in Fig. 8b, d. Using the identical criteria, the sample can be safely seen as negative at the end of the 35^th^ cycle. Besides end-point values illustrated here, more data in sequence are presented in Fig. S4.

Theoretically, considering the definition of baseline and threshold by conventional qPCR analysis [41], the Cq value can be easily affected by the parameters set by the operator or software of the instrument. It should be worth noting that the Cq value has shown inconsistency among assays most recently [42]. Furthermore, it is difficult for the conventional qPCR to calculate Cq during reaction in a real-time manner. Finally, the existing laboratory or clinical qPCR tests usually output Cq value on the scale of a whole test without automatic discrimination of individual reactions. By contrast, the AI-aided method can perform intuitive and accurate real-time analytics promoting a novel paradigm of qPCR analysis independent of Cq. Also, the method is capable of forecasting the final output of qPCR and the trend of amplification curves before end-point Cq calculation. Most importantly, the prediction method fully explored the dynamics or signal features of each reaction, and thus this theoretical innovation will assist scientists and physicians to evaluate the individual variation.

### 3.5. Parameter assessment

**Fig. 9:**
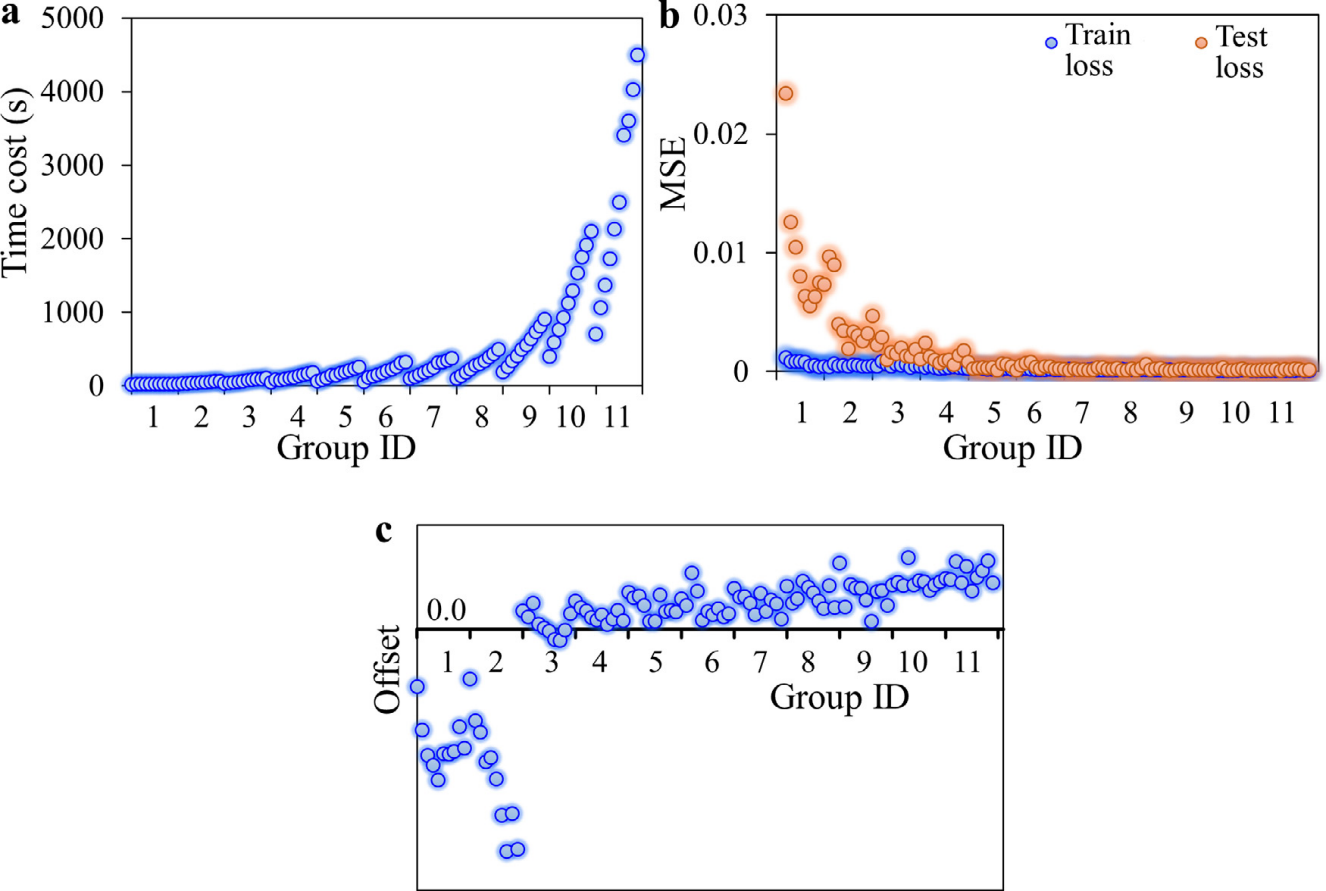
**Parameter assessment tests.** (a) Comparison study of time cost by 11 groups using gate recurrent unit (GRU) method. (b) Comparison study of train and test loss by 11 groups using GRU. (c) Comparison study of deviations of end-point output by 11 groups using GRU.

Currently, there are few standardizations and guides on hyperparameter tuning for AI methods. We observed the number of interpolated data and the input length of the data series affected the calculation speed and accuracy most significantly. Thus, we performed a trial process to further evaluate the chosen parameters of the GRU-based neural network. Parameter setting details are listed in Table S3. In brief, 11 data groups were built containing the number of interpolated data ranging from 2 to 600. For each interpolation, various input lengths ranging from 1 to 10 were used. The time cost of a single run, train and test loss, and variance between true data and the predicted at the end-point were studied among the groups (Fig. 9).

With interpolation data increasing, time consumption for each forecasting run also increased and became more apparent for group #8. Within a group, more input data also takes more computing power to process. On the other hand, the MSE value decreased with increasing data quantity, especially for the test process. This indicates an improved training and validation accuracy by using a larger dataset. Then, offsets between true data and predicated value became optimized at group #3, which included 10 interpolation data with input lengths of 4 or 5. The offset started to slowly rise with dataset capacity after group #3. The results consolidate the parameter setting of the neural network by which the run time for a single PCR test was 50 to 57 s, which was comparable to a PCR cycle. Notably, the time cost can be decreased further by gathering more computing resources.

Finally, we investigated the overfitting issue based on the selected GRU model. The impact of input cycle number (i.e. data for training and testing) on overfitting in the deep learning model was studied. MSE values at the 40^th^ epoch on loss function curves of the 83 samples were plotted against input cycle numbers as shown in Fig. S5. Overall, both the training loss and validation loss decreased with the cycle number used for prediction. Using data by 10 or 12 qPCR cycles, the training loss is much lower than validation loss by an order of magnitude. In this case, overfitting may exist and the prediction results had low accuracy. When cycle numbers increased to between 14 and 18, variations between the two-loss functions reduced to the same magnitude except for a few outliers. Then, training loss became similar to but slightly lower than validation loss when data included 20 cycles or more. Also, in this scenario, values of outliers were restricted below 0.001. Considering qPCR signals observed by this work were on the magnitude of 1000 (Fig. 3a) and the data normalization process, the overfitting had been effectively restrained by the parameters settings.

## 4. Conclusion

This work leverages various methodologies from multiple disciplines, including precision manufacturing, instrument technology, molecular detection, and bioinformatics, to provide perspectives and insights beyond the scope of a single scientific area. A novel AI-aided on-chip approach to detect RNA templates of the SARS-CoV-2 ORF1ab gene target was presented. µPADs that are compatible with the commercial qPCR machine were developed for on-chip data acquisition. qPCR data were delivered to three deep neural networks consisting of stacked RNN, LSTM, and GRU. GRU had the best performance in terms of accuracy and earliness. Qualitative forecasting became available as early as 13 cycles indicating an improvement of PCR testing efficiency of 67.5% as the turnaround time was reduced to 12 min. Accurate prediction of end-point value and dynamic trend of qPCR curves were obtained by GRU around the 20^th^ cycle. The mean absolute percentage error by the GRU model was 2.1%. Additionally, the model parameter assessment study indicated that prediction accuracy improved along with the number of datasets. We also empirically proposed a calculation method for obtaining a critical cycle for quantitative analysis of intra-assay. The presented approach was the first to integrate AI for on-chip qPCR data analysis and it enabled novel predictive analytics for the diagnosis of infectious diseases. The approach was capable of forecasting the final output and trend of qPCR independent of end-point Cq calculation but fully exploring the dynamics or intrinsic features of each reaction. This innovation may assist the whole society to accelerate the response to novel disease outbreaks. AI-aided analytics is universally applicable and can be extended to multiple areas of fundamental research. Nowadays, point-of-care testing (POCT) and personalized medicine (PM) are becoming more realistic with the growth of new diagnostic and informatics methods. In the future, integration of AI-aided diagnosis, POCT, PM with the internet of things (IoT) concept could be valuable to pursue.

## Declaration of Competing Interest

The authors declare that they have no conflicts of interest in this work.

## Appendix Supplementary materials

Image, application 1
