# Supplementary material for: AI-aided on-chip nucleic acid assay for smart diagnosis of infectious disease
Source: Fundam Res. 2021 Dec 28;2(3):476–86. doi: 10.1016/j.fmre.2021.12.005 (PMC8712671; doi:10.1016/j.fmre.2021.12.005)
Supplement: Supplementary file 1 [file mmc1.docx]

Supplementary Material

**AI-aided on-chip Nucleic Acid Assay for Smart Diagnosis of Infectious Disease**

Hao Sun^a^, Linghu Xiong^a^, Yi Huang^b, c^, Xinkai Chen^a^, Yongjian Yu^a^, Shaozhen Ye^d, e^, Hui Dong^a, f*^, Yuan Jia^g*^, Wenwei Zhang^h*^

^a^ School of Mechanical Engineering and Automation, Fuzhou University, Fuzhou, 350116, China

^b^ Provincial Clinical College, Fujian Medical University, Fuzhou 350001, China

^c^ Center for Experimental Research in Clinical Medicine, Fujian Provincial Hospital, Fuzhou 350001, China.

^d^ College of Mathematics and Computer Science, Fuzhou University, Fuzhou, 350116, China

^e^ Institute of Intelligent Manufacturing and Simulation, Fuzhou University, Fuzhou, 350116, China

^f^ Fujian Provincial Collaborative Innovation Center of High-End Equipment Manufacturing, Fuzhou, 350001, China

^g^ College of New Materials and New Energies, Shenzhen Technology University, Shenzhen, 518118, China

^h^ Sino-German College of Intelligent Manufacturing, Shenzhen Technology University, Shenzhen, 518118, China


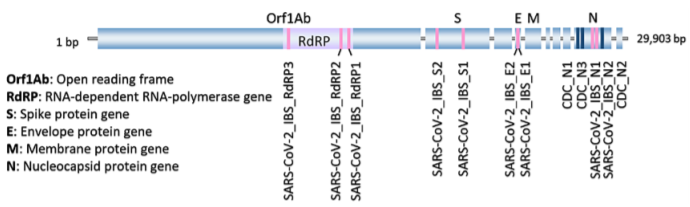


Figure 1. Gene structure of SARS‑CoV‑2^1^


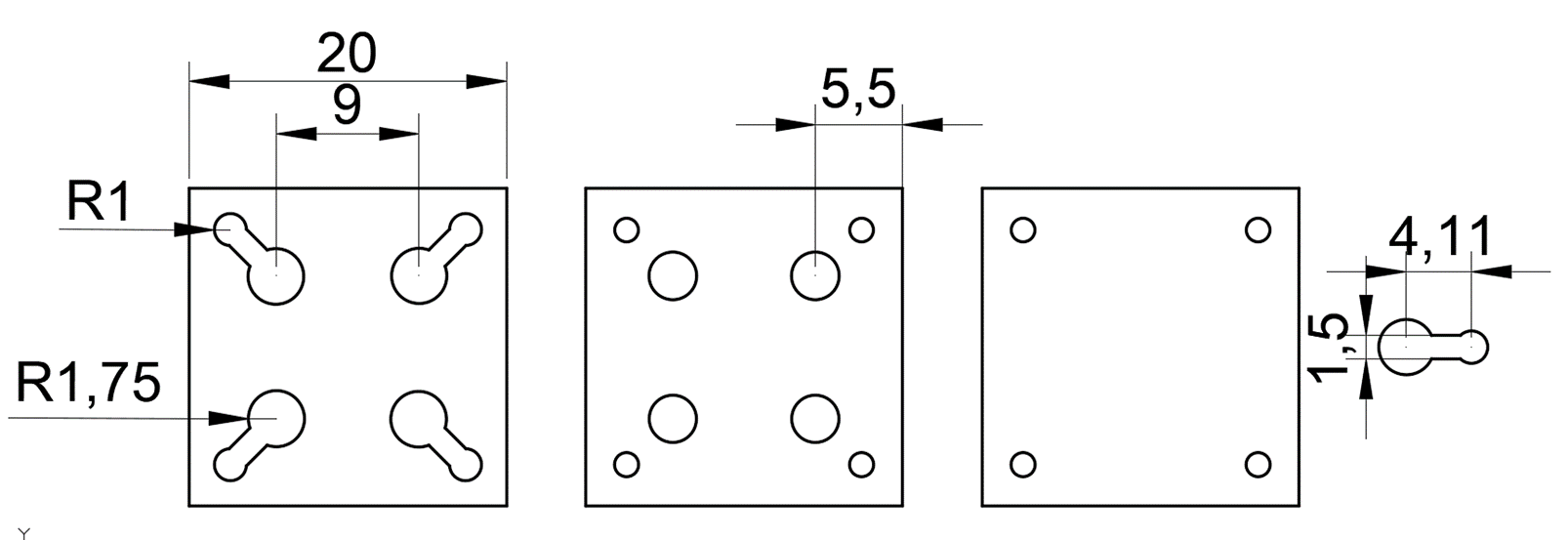


Figure 2. µPAD design layout

Table 1. Material cost of µPAD

|  | Price/area | Area per chip | Price per chip |
| --- | --- | --- | --- |
| Glass slide | 0.2357 $/400mm² | 400mm² | 0.2357 $ |
| non-transparent PVC | 0.0911 $/66000mm² | 1600mm² | 0.0022 $ |
| PMMA | 0.0754 $/62370mm² | 400mm² | 0.0005 $ |
| Thin film PVC with thermosensitive gel | 0.0236 $/62371mm² | 400mm² | 0.0002 $ |
| Filter paper | 0.1571 $/6361mm² | 100mm² | 0.0003 $ |
| Total cost per chip |  | 0.2389 $ |  |


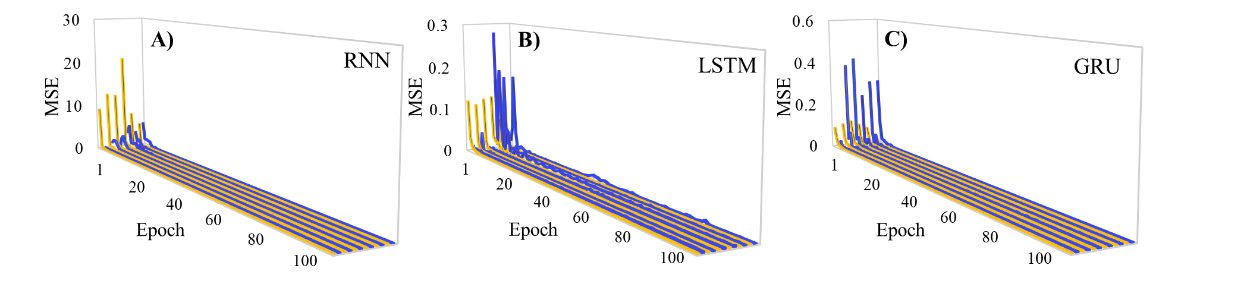


Figure 3. Train and test loss curves using A) RNN; B) LSTM; C) GRU.

Table 2. Prediction accuracy obtained using MAPE, MAE and SMAPE

|  | MAE | MAPE | SMAPE |
| --- | --- | --- | --- |
| GRU_NC#1 | 129.0489982 | 29.0972097 | 47.95788563 |
| GRU_NC#2 | 75.34341499 | 3.596620204 | 3.749259701 |
| GRU_NC#3 | 51.14516398 | 13.25194748 | 13.4849938 |
| GRU_sample#1 | 72.82438723 | 3.569066668 | 3.420331042 |
| GRU_sample#2 | 27.16240823 | 1.179530475 | 1.189126735 |
| GRU_sample#3 | 41.79502709 | 1.653799819 | 1.632441182 |
| LSTM_NC#1 | 129.6698828 | 29.18044221 | 48.30872449 |
| LSTM_NC#2 | 46.15989597 | 2.248603729 | 2.311283443 |
| LSTM_NC#3 | 61.66448821 | 14.98240803 | 15.58194265 |
| LSTM_sample#1 | 345.2973501 | 13.76715286 | 16.81443363 |
| LSTM_sample#2 | 230.697618 | 8.584756343 | 7.722991466 |
| LSTM_sample#3 | 439.1897545 | 7.913129201 | 8.723390822 |
| RNN_NC#1 | 132.469175 | 29.53189056 | 49.71443668 |
| RNN_NC#2 | 210.5750113 | 9.470885421 | 10.92867347 |
| RNN_NC#3 | 60.45760209 | 14.78522949 | 15.33596733 |
| RNN_sample#1 | 505.1539133 | 20.4689205 | 27.64774668 |
| RNN_sample#2 | 479.7485267 | 14.75652349 | 18.60798109 |
| RNN_sample#3 | 562.5915344 | 9.943576127 | 11.32798421 |


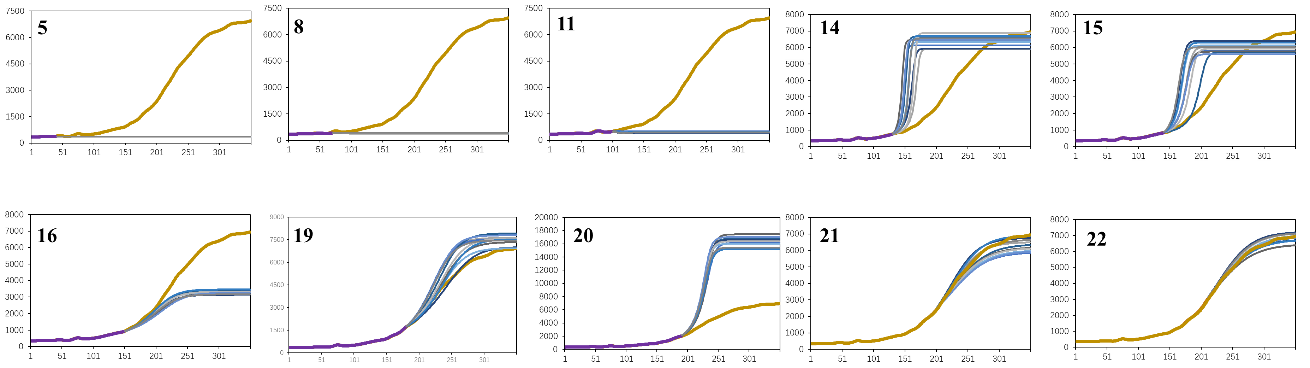


Figure 4. Cycle-dependent prediction. The cycle numbers for model training and testing are shown in the upper left corner.


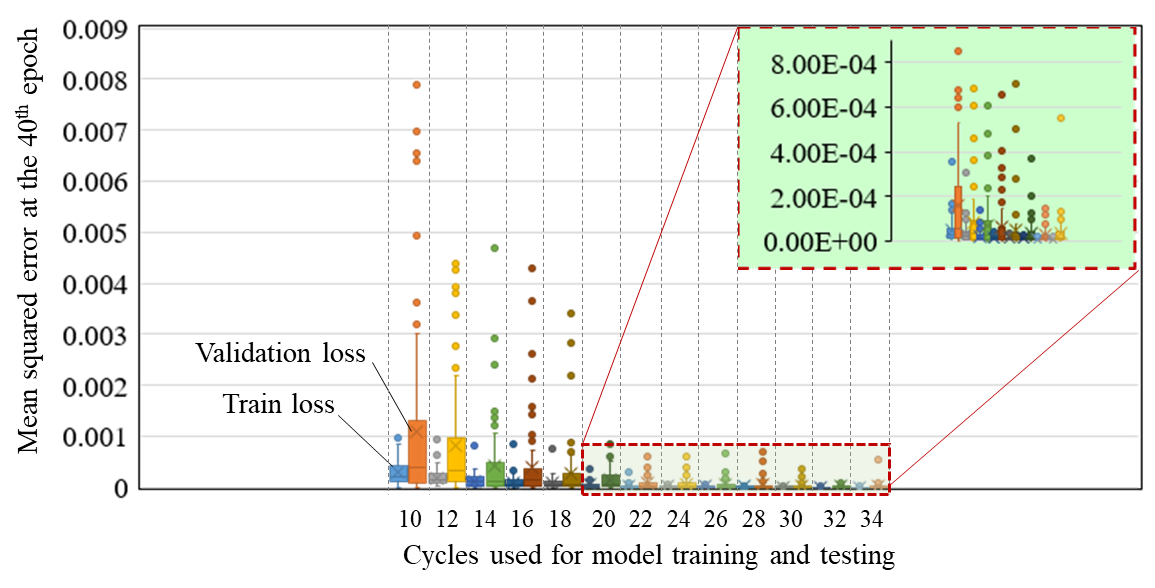


Figure 5. Box-plots of loss function obtained by 83 sample tests depicting the effect of input cycle number on overfitting

Table 3. Data from the parameter assessment study

| **Group** | **Inter. data** | **Win. size** | **True value** | **Pred. value** | **Variance** | **Computing time (s)** | **Train loss** | **Validation loss** | **Ratio** |
| --- | --- | --- | --- | --- | --- | --- | --- | --- | --- |
| 1 | 2 | 1 | 6946 | 12477 | -5531 | 12 | 0.001105 | 0.023369863 | 0.85 |
|  | 2 | 2 | 6946 | 16645 | -9699 | 15 | 0.000787 | 0.012520892 | 0.85 |
|  | 2 | 3 | 6946 | 19043 | -12097 | 15 | 0.000778 | 0.010430985 | 0.85 |
|  | 2 | 4 | 6946 | 19986 | -13040 | 16 | 0.000821 | 0.007959061 | 0.85 |
|  | 2 | 5 | 6946 | 21407 | -14461 | 17 | 0.000744 | 0.006316716 | 0.85 |
|  | 2 | 6 | 6946 | 18912 | -11966 | 18 | 0.000414 | 0.00548691 | 0.8 |
|  | 2 | 7 | 6946 | 18923 | -11977 | 20 | 0.00047 | 0.006265853 | 0.8 |
|  | 2 | 8 | 6946 | 18672 | -11726 | 19 | 0.000332 | 0.007434731 | 0.75 |
|  | 2 | 9 | 6946 | 16329 | -9383 | 20 | 0.000365 | 0.007280486 | 0.75 |
|  | 2 | 10 | 6946 | 18384 | -11438 | 18 | 0.000328 | 0.009641468 | 0.7 |
| 2 | 5 | 1 | 6946 | 11730 | -4784 | 19 | 0.000635 | 0.008928671 | 0.85 |
|  | 5 | 2 | 6946 | 15731 | -8785 | 24 | 0.000441 | 0.003958205 | 0.85 |
|  | 5 | 3 | 6946 | 16854 | -9908 | 28 | 0.000496 | 0.003381046 | 0.85 |
|  | 5 | 4 | 6946 | 19680 | -12734 | 32 | 0.000376 | 0.00185828 | 0.85 |
|  | 5 | 5 | 6946 | 19273 | -12327 | 36 | 0.000515 | 0.003318688 | 0.85 |
|  | 5 | 6 | 6946 | 21329 | -14383 | 40 | 0.000492 | 0.002984631 | 0.85 |
|  | 5 | 7 | 6946 | 24782 | -17836 | 43 | 0.000391 | 0.002526874 | 0.85 |
|  | 5 | 8 | 6946 | 28258 | -21312 | 48 | 0.000395 | 0.003148795 | 0.85 |
|  | 5 | 9 | 6946 | 24618 | -17672 | 54 | 0.000439 | 0.004648462 | 0.85 |
|  | 5 | 10 | 6946 | 28072 | -21126 | 57 | 0.000401 | 0.002175356 | 0.85 |
| 3 | 10 | 1 | 6946 | 5187 | 1759 | 26 | 0.000796 | 0.002856139 | 0.95 |
|  | 10 | 2 | 6946 | 5775 | 1171 | 34 | 0.000571 | 0.000937793 | 0.95 |
|  | 10 | 3 | 6946 | 4453 | 2493 | 42 | 0.000399 | 0.001573053 | 0.95 |
|  | **10** | **4** | **6946** | **6476** | **470** | **50** | **0.000581** | **0.001459293** | **0.95** |
|  | **10** | **5** | **6946** | **6860** | **86** | **57** | **0.0005** | **0.001951299** | **0.95** |
|  | 10 | 6 | 6946 | 7140 | -194 | 67 | 0.000588 | 0.001278451 | 0.95 |
|  | 10 | 7 | 6946 | 7944 | -998 | 74 | 0.000295 | 0.001232538 | 0.95 |
|  | 10 | 8 | 6946 | 8027 | -1081 | 82 | 0.000429 | 0.001806736 | 0.95 |
|  | 10 | 9 | 6946 | 7053 | -107 | 87 | 0.000364 | 0.000997883 | 0.95 |
|  | 10 | 10 | 6946 | 5467 | 1479 | 99 | 0.000285 | 0.002389635 | 0.9 |
| 4 | 20 | 1 | 6946 | 4248 | 2698 | 39 | 0.000377 | 0.001196099 | 0.95 |
|  | 20 | 2 | 6946 | 4898 | 2048 | 76 | 0.000278 | 0.000920103 | 0.95 |
|  | 20 | 3 | 6946 | 5215 | 1731 | 71 | 0.000194 | 0.000675421 | 0.95 |
|  | 20 | 4 | 6946 | 5836 | 1110 | 84 | 0.000233 | 0.000914868 | 0.95 |
|  | 20 | 5 | 6946 | 6108 | 838 | 98 | 0.00028 | 0.000934889 | 0.95 |
|  | 20 | 6 | 6946 | 5598 | 1348 | 110 | 0.00036 | 0.000502644 | 0.95 |
|  | 20 | 7 | 6946 | 6536 | 410 | 128 | 0.000272 | 0.00131277 | 0.95 |
|  | 20 | 8 | 6946 | 6011 | 935 | 143 | 0.000239 | 0.001717905 | 0.95 |
|  | 20 | 9 | 6946 | 5168 | 1778 | 157 | 0.000177 | 0.000732126 | 0.95 |
|  | 20 | 10 | 6946 | 6137 | 809 | 175 | 0.000212 | 0.000212275 | 0.95 |
| 5 | 30 | 1 | 6946 | 3424 | 3522 | 53 | 0.000263 | 0.000226951 | 0.95 |
|  | 30 | 2 | 6946 | 3946 | 3000 | 77 | 0.000187 | 0.000276324 | 0.95 |
|  | 30 | 3 | 6946 | 3787 | 3159 | 100 | 0.000164 | 0.000229736 | 0.95 |
|  | 30 | 4 | 6946 | 4692 | 2254 | 137 | 0.000166 | 0.000269654 | 0.95 |
|  | 30 | 5 | 6946 | 6207 | 739 | 140 | 0.000234 | 6.42567E-05 | 0.95 |
|  | 30 | 6 | 6946 | 6193 | 753 | 164 | 0.000132 | 0.000596384 | 0.95 |
|  | 30 | 7 | 6946 | 3710 | 3236 | 183 | 0.000199 | 0.000581893 | 0.95 |
|  | 30 | 8 | 6946 | 5267 | 1679 | 204 | 0.000139 | 0.000369173 | 0.95 |
|  | 30 | 9 | 6946 | 5144 | 1802 | 224 | 0.000244 | 4.13525E-05 | 0.95 |
|  | 30 | 10 | 6946 | 5315 | 1631 | 247 | 0.000141 | 0.000487815 | 0.95 |
| 6 | 40 | 1 | 6946 | 4033 | 2913 | 45 | 0.000215 | 0.000684353 | 0.95 |
|  | 40 | 2 | 6946 | 4669 | 2277 | 106 | 0.000139 | 0.000769674 | 0.95 |
|  | 40 | 3 | 6946 | 1596 | 5350 | 123 | 0.000117 | 0.000317832 | 0.95 |
|  | 40 | 4 | 6946 | 3313 | 3633 | 146 | 0.000127 | 0.000221063 | 0.95 |
|  | 40 | 5 | 6946 | 6123 | 823 | 171 | 0.000111 | 0.000382014 | 0.95 |
|  | 40 | 6 | 6946 | 5237 | 1709 | 198 | 0.000136 | 0.000377402 | 0.95 |
|  | 40 | 7 | 6946 | 5568 | 1378 | 223 | 0.000101 | 0.000190437 | 0.95 |
|  | 40 | 8 | 6946 | 5020 | 1926 | 251 | 0.000101 | 0.000205664 | 0.95 |
|  | 40 | 9 | 6946 | 5776 | 1170 | 299 | 9.83E-05 | 0.000147177 | 0.95 |
|  | 40 | 10 | 6946 | 5452 | 1494 | 318 | 0.000133 | 3.9362E-05 | 0.95 |
| 7 | 50 | 1 | 6946 | 3049 | 3897 | 92 | 0.000162 | 0.000198614 | 0.95 |
|  | 50 | 2 | 6946 | 3891 | 3055 | 115 | 0.000115 | 0.000106609 | 0.95 |
|  | 50 | 3 | 6946 | 3819 | 3127 | 143 | 0.000101 | 0.000129735 | 0.95 |
|  | 50 | 4 | 6946 | 4489 | 2457 | 177 | 0.000121 | 6.43725E-05 | 0.95 |
|  | 50 | 5 | 6946 | 5574 | 1372 | 206 | 0.000132 | 9.00964E-05 | 0.95 |
|  | 50 | 6 | 6946 | 3533 | 3413 | 240 | 8E-05 | 0.000276784 | 0.95 |
|  | 50 | 7 | 6946 | 5264 | 1682 | 306 | 7.17E-05 | 0.000311323 | 0.95 |
|  | 50 | 8 | 6946 | 4146 | 2800 | 317 | 6.74E-05 | 0.000159411 | 0.95 |
|  | 50 | 9 | 6946 | 4541 | 2405 | 339 | 5.83E-05 | 0.000168735 | 0.95 |
|  | 50 | 10 | 6946 | 6001 | 945 | 366 | 8.33E-05 | 0.000225037 | 0.95 |
| 8 | 60 | 1 | 6946 | 2866 | 4080 | 95 | 0.000138 | 7.40112E-05 | 0.95 |
|  | 60 | 2 | 6946 | 4463 | 2483 | 133 | 0.000109 | 7.67493E-05 | 0.95 |
|  | 60 | 3 | 6946 | 4078 | 2868 | 174 | 0.000116 | 0.000213607 | 0.95 |
|  | 60 | 4 | 6946 | 2349 | 4597 | 222 | 8.22E-05 | 4.81534E-05 | 0.95 |
|  | 60 | 5 | 6946 | 2923 | 4023 | 269 | 9.53E-05 | 0.000249256 | 0.95 |
|  | 60 | 6 | 6946 | 3489 | 3457 | 295 | 0.000106 | 0.000581127 | 0.95 |
|  | 60 | 7 | 6946 | 4248 | 2698 | 333 | 6.37E-05 | 0.000126553 | 0.95 |
|  | 60 | 8 | 6946 | 4977 | 1969 | 385 | 6.04E-05 | 0.000182576 | 0.95 |
|  | 60 | 9 | 6946 | 2814 | 4132 | 430 | 5.77E-05 | 0.000243771 | 0.95 |
|  | 60 | 10 | 6946 | 4875 | 2071 | 491 | 9.39E-05 | 6.94679E-05 | 0.95 |
| 9 | 120 | 1 | 6946 | 637 | 6309 | 179 | 8.63E-05 | 1.88468E-05 | 0.95 |
|  | 120 | 2 | 6946 | 4864 | 2082 | 252 | 4.79E-05 | 3.88616E-05 | 0.95 |
|  | 120 | 3 | 6946 | 2694 | 4252 | 335 | 5.16E-05 | 0.000166358 | 0.95 |
|  | 120 | 4 | 6946 | 3008 | 3938 | 400 | 5.6E-05 | 0.00021127 | 0.95 |
|  | 120 | 5 | 6946 | 3029 | 3917 | 485 | 7.14E-05 | 0.000114324 | 0.95 |
|  | 120 | 6 | 6946 | 4139 | 2807 | 545 | 5.26E-05 | 8.31299E-05 | 0.95 |
|  | 120 | 7 | 6946 | 6210 | 736 | 632 | 3.89E-05 | 9.90654E-05 | 0.95 |
|  | 120 | 8 | 6946 | 3347 | 3599 | 724 | 5.46E-05 | 5.68373E-05 | 0.95 |
|  | 120 | 9 | 6946 | 3238 | 3708 | 801 | 5.29E-05 | 0.000121785 | 0.95 |
|  | 120 | 10 | 6946 | 4665 | 2281 | 901 | 4.43E-05 | 4.98101E-05 | 0.95 |
| 10 | 300 | 1 | 6946 | 2719 | 4227 | 393 | 2.84E-05 | 0.000170252 | 0.95 |
|  | 300 | 2 | 6946 | 2490 | 4456 | 583 | 2.46E-05 | 0.000271232 | 0.95 |
|  | 300 | 3 | 6946 | 2770 | 4176 | 760 | 2.72E-05 | 6.97784E-05 | 0.95 |
|  | 300 | 4 | 6946 | 119 | 6827 | 921 | 2.59E-05 | 5.16704E-05 | 0.95 |
|  | 300 | 5 | 6946 | 2752 | 4194 | 1114 | 3.2E-05 | 0.000116656 | 0.95 |
|  | 300 | 6 | 6946 | 2332 | 4614 | 1290 | 4.26E-05 | 0.000194412 | 0.95 |
|  | 300 | 7 | 6946 | 2434 | 4512 | 1528 | 6.34E-05 | 6.25126E-05 | 0.95 |
|  | 300 | 8 | 6946 | 3283 | 3663 | 1740 | 2.53E-05 | 6.03299E-05 | 0.95 |
|  | 300 | 9 | 6946 | 2757 | 4189 | 1907 | 2.73E-05 | 8.4021E-05 | 0.95 |
|  | 300 | 10 | 6946 | 2405 | 4541 | 2095 | 2.78E-05 | 9.69014E-05 | 0.95 |
| 11 | 600 | 1 | 6946 | 2127 | 4819 | 697 | 1.56E-05 | 0.000115053 | 0.95 |
|  | 600 | 2 | 6946 | 2227 | 4719 | 1058 | 1.82E-05 | 0.000107773 | 0.95 |
|  | 600 | 3 | 6946 | 460 | 6486 | 1362 | 2.62E-05 | 0.000132918 | 0.95 |
|  | 600 | 4 | 6946 | 2543 | 4403 | 1720 | 1.72E-05 | 3.71195E-05 | 0.95 |
|  | 600 | 5 | 6946 | 972 | 5974 | 2125 | 2.51E-05 | 0.000173641 | 0.95 |
|  | 600 | 6 | 6946 | 3325 | 3621 | 2492 | 2.82E-05 | 0.000121036 | 0.95 |
|  | 600 | 7 | 6946 | 2012 | 4934 | 3396 | 2.14E-05 | 0.000210731 | 0.95 |
|  | 600 | 8 | 6946 | 1369 | 5577 | 3594 | 4.98E-05 | 0.000132799 | 0.95 |
|  | 600 | 9 | 6946 | 406 | 6540 | 4019 | 2.13E-05 | 6.91307E-05 | 0.95 |
|  | 600 | 10 | 6946 | 2517 | 4429 | 4491 | 2.33E-05 | 9.9043E-05 | 0.95 |

**Reference**

[1] Falzone L, Musso N, Gattuso G, et al. Sensitivity assessment of droplet digital PCR for SARS-CoV-2 detection. International journal of molecular medicine, 2020, 46(3): 957-964.
